# A Case of Carcinoma of the Parturient Uterus, Removed Three Days after Confinement—Recovery*Read before the Southern Surgical and Gynæcological Association, November 14, 1894, at Charleston, S. C.

**Published:** 1895-02

**Authors:** George H. Noble

**Affiliations:** Atlanta, Ga., Gynæcologist to Grady Hospital


### A CASE OF CARCINOMA OF THE PARTURIENT UTERUS, REMOVED THREE DAYS AFTER CONFINEMENT.—RECOVERY.*

By GEORGE H. NOBLE, M. D., Atlanta, Ga.,

Gynæcologist to Grady Hospital.

[*From American Gynæcological and Obstetrical Journal.*]

This specimen is one of carcinoma of the parturient uterus, removed by vaginal hysterectomy three days after labor. This woman had previously been confined, sustaining a laceration of the cervix uteri, which perhaps was a factor in the cause of the disease. In the first few months of the last pregnancy, she was treated locally by her family physician; but there was nothing, I am told, to cause a suspicion of malignancy. The treatment was suspended when quickening became perceptible, but it was soon followed by offensive watery discharges admixed with pus and blood.

Dr. W. B. Parks, who was attending her at this time, recognized the character of the case and asked me to see her on the 4th day of last June. He stated that she had been in labor for some time before he saw her, altogether a little more than twenty-four hours. She was very much exhausted; pulse 135 per minute, very weak and compressible, with marked anæmia; the temperature 102°. The history gave a clear evidence of repeated chills or rigors with varying temperature and excessive diaphoresis for three months past, and in that time she was markedly reduced in flesh and strength.

Almost the entire vaginal portion of the cervix was destroyed, less than one-fourth of its circumference remaining intact. The induration extended deep into the uterine tissue but could not be felt beyond the limits of that organ. The roughened ulcerated surface was easily traced for a considerable distance within the cervix, the os being dilated to about five centimetres in diameter. Her condition was unpromising and surgical interference was clearly interdicted, so the os and vagina were cleansed thoroughly and lightly dressed with gauze She was then placed profoundly under the

*Read before the Southern Surgical and Gynæcological Association, November 14, 1894, at Charleston, S. C.

influence of morphia sulphate, with a view of arresting labor, securing rest and recuperation sufficient to permit evacuation of the uterus, which occurred spontaneously twelve hours later. The child was poorly nourished and lived only a few weeks, finally dying of inanition. A slight laceration occurred in the delivery, the rent taking place in the unulcerated portion of the cervix, but not implicating the vagina. The surface was daily curetted and dressed antiseptically up to the time of the operation, a noticeable effect of which was the decrease in temperature, the elevation not going over 0. 5°.

The operation was deferred a few days for the purpose of securing some reaction in the patient that she might be enabled to undergo the ordeal and to effect through the process of involution some reduction in the size of the uterus. For three days after delivery the pulse ranged from 100 to 120 per minute, quite weak, but slightly improved in volume, with temperature a little more than normal (99° to 99.5° degrees). Her condition was not encouraging even under vigorous stimulation by the mouth and by hypodermic use of digitaline, strychnine atropine, etc. The ulceration was so deep at one point that it nearly penetrated the cervical walls, and as she had been so profoundly impressed by septic absorption it was thought best to avoid further delay in operating, lest the rapid extension of the disease and sepsis should put her beyond surgical relief. After careful antiseptic preparation the uterus was packed with gauze, the ragged edges of the cervix trimmed away the os rolled in upon itself and closed. The successive steps of the operation then followed:

The greatly dilated vessels attending the gravid state had not been sufficiently reduced in size to relieve the increased dangers of hemorrhage, consequently compression forceps were in demand at each step or turn in the operation. An attempt was made to tie off the broad ligaments, but the parts were so swelled and puffy that it was found impracticable after the lower section was ligated. The uterus was so large that it well filled the pelvis so that no room was left to manipulate the ligatures, only space enough for two fingers being available, consequently clamps were employed to complete the work. Morcellation or segmentation was rejected in this case, the extremely anæmic condition of the patient forbidding any unnecessary loss of blood.

On the left side two, and upon the right three clamps were placed above the ligatures and about a dozen artery forceps were used in other parts of the wound, as time would not allow ligation of bleeding points. Separation of the bladder attachments was tedious on account of the extended surface rendering the peritoneal fold rather difficult of access. Douglas' pouch was thickened by old adhesions, the point of penetration being at least three and a half centimetres thick. There was also a very dense adhesion of the descending colon to the posterior uterine surface, about seven centimetres in diameter.

The peritoneum and mucous membrane over the bladder was approximated by compression forceps and the wound drained with gauze. She bore the operation well up to the time of cutting the uterus away, when it became necessary to readjust the clamp upon the right uterine artery as there was a small amount of uterine tissue in the stump and in order to remove it the clamp required setting back to the ureter. There was no excessive hemorrhage, but what blood did escape was more than she could well spare; for a time the radial pulse was imperceptible after returning her to the bed. I directed the infusion of the normal salt solution which was skillfully administered by my associate, Dr. Taliaferro, with the happiest results.

The clamps were removed in forty-eight hours, excepting the one upon the right uterine artery. In endeavoring to disengage the instrument free hemorrhage occurred; it was instantly closed and left for twelve hours more when it was successfully taken away.

There was no evidence of sepsis, but the heart's action was weak requiring hypodermic stimulation for four or five days. The stumps sloughed off and the wound closed by granulation, except at a point on the left side where a very small ureteral fistula occurred, which closed spontaneously in eight weeks. The woman made a satisfactory recovery and visited Virginia in twelve weeks.

The questions naturally arise: What is the advantage of hysterectomy over Porro's operation, and if hysterectomy is preferable should the vaginal or abdominal method be given precedence over the other? To the first I would answer that hysterectomy undoubtedly promises more to the mother than Porro's operation in cases where the disease is confined to the uterus, and I assert that when the cancerous mass can be successfully removed, it is the duty of the surgeon to do it, as Porro's method merely bridges the woman over the puerperal state and leaves her to her fate. In radical removal there is a promise of cure. In answer to the second question, it is evident that the method of operating must depend largely upon the character of each individual case; thus the vaginal operation may be done when it is desirable to take advantage of the diminished liability to shock, even though the large size of the uterus may render the operation more tedious. Removal by segmentation or morcellation will greatly expedite the work in such instances, but it entails the loss of more blood, that can illy be spared by women worn down by rapidly extending malignant disease, which is always the case in cancer of the gravid uterus. And it exposes the peritoneum to infection from the uterine cavity, which may not be perfectly sterilized even in the hands of very cautious persons.

In hysterectomy by the abdominal method in Trendelenburg's position it is claimed (Wyley, Polk and others) that the advantage of rapidity in work and the ability to remove more thoroughly the diseased portions even where the vagina is involved, is of inestimable value, but there is greater danger of shock. If the disease involves the vagina to such an extent that the fœtus can not be delivered *per vias naturales*, hysterectomy is out of the question and the case falls to the last resort, the Porro operation, with the hope of saving the life of the child and diminishing the risk of sepis in the mother.

The mortality in Porro's operation is according to Harris (*New York Journal of Gynæcology and Obstetrics*, vol. iii, No. 4, p. 273), from eight to twelve per cent. under the most favorable circumstances and in the hands of experienced operators. In vaginal hysterectomy for bilateral disease, according to Jacobs (*Mod. Med.*, vol. iii, No. 6, p 143)* it has in 690 cases been reduced to 4.49 per cent. and in 184 of his individual

*Also *American Journal of Obstetrics*, Nov., 1894, vol. xxx, p. 648.

cases to 2.7 per cent., thus showing a much less mortality under ordinary circumstances in favor of the latter operation.

In the face of this showing it is yet a question whether the abdominal method with its advantages and disadvantages should be given preference over the vaginal operation, which is attended with less shock. Wyley says: "Though the mortality from vaginal hysterectomy is very small, complete removal by cœliotomy is, I believe, the operation of the future, as more of the diseased tissue in the broad ligaments and vagina can be removed than by vaginal hysterectomy."

However, it is not my purpose to point out the advantage of one operation over the other, but to show the feasibility of hysterectomy in the puerperal state for cancer of the uterus, as this case clearly demonstrates, even though it is too early in this instance to claim immunity from return of the disease.

